# Systemic RNAi mediated gene silencing in the anhydrobiotic nematode *Panagrolaimus superbus*

**DOI:** 10.1186/1471-2199-9-58

**Published:** 2008-06-19

**Authors:** Adam J Shannon, Trevor Tyson, Ilona Dix, Jacqueline Boyd, Ann M Burnell

**Affiliations:** 1 Biology Department, National University of Ireland, Maynooth, Co. Kildare, Ireland; 2 School of Animal, Rural and Environmental Sciences, Nottingham Trent University, Nottingham Rd., Southwell, NG25 0QF, UK

## Abstract

**Background:**

Gene silencing by RNA interference (RNAi) is a powerful tool for functional genomics. Although RNAi was first described in *Caenorhabditis elegans*, several nematode species are unable to mount an RNAi response when exposed to exogenous double stranded RNA (dsRNA). These include the satellite model organisms *Pristionchus pacificus *and *Oscheius tipulae*. Available data also suggest that the RNAi pathway targeting exogenous dsRNA may not be fully functional in some animal parasitic nematodes. The genus *Panagrolaimus *contains bacterial feeding nematodes which occupy a diversity of niches ranging from polar, temperate and semi-arid soils to terrestrial mosses. Thus many *Panagrolaimus *species are adapted to tolerate freezing and desiccation and are excellent systems to study the molecular basis of environmental stress tolerance. We investigated whether *Panagrolaimus *is susceptible to RNAi to determine whether this nematode could be used in large scale RNAi studies in functional genomics.

**Results:**

We studied two species: *Panagrolaimus *sp. PS1159 and *Panagrolaimus superbus*. Both nematode species displayed embryonic lethal RNAi phenotypes following ingestion of *Escherichia coli *expressing dsRNA for the *C. elegans *embryonic lethal genes *Ce-lmn-1 *and *Ce-ran-4*. Embryonic lethal RNAi phenotypes were also obtained in both species upon ingestion of dsRNA for the *Panagrolaimus *genes *ef1b *and *rps-2*. Single nematode RT-PCR showed that a significant reduction in mRNA transcript levels occurred for the target *ef1b *and *rps-2 *genes in RNAi treated *Panagrolaimus *sp. 1159 nematodes. Visible RNAi phenotypes were also observed when *P. superbus *was exposed to dsRNA for structural genes encoding contractile proteins. All RNAi phenotypes were highly penetrant, particularly in *P. superbus*.

**Conclusion:**

This demonstration that *Panagrolaimus *is amenable to RNAi by feeding will allow the development of high throughput methods of RNAi screening for *P. superbus*. This greatly enhances the utility of this nematode as a model system for the study of the molecular biology of anhydrobiosis and cryobiosis and as a possible satellite model nematode for comparative and functional genomics. Our data also identify another nematode infraorder which is amenable to RNAi and provide additional information on the diversity of RNAi phenotypes in nematodes.

## Background

RNA interference (RNAi) was first described in the nematode *Caenorhabditis elegans *[1,2], when it was found that the injection of double-stranded RNA (dsRNA) into a hermaphrodite worm resulted in the degradation of endogenous mRNA corresponding in sequence to the injected dsRNA. This resulted in a loss of function phenotype for the target gene. Potent RNAi effects can also be achieved in *C. elegans *by soaking the nematodes in a dsRNA solution [3] or by feeding the nematodes on bacteria engineered to express dsRNA of the target gene [4]. The potential for RNAi by feeding to be used in a large scale manner for functional genomics was demonstrated by the construction of a feeding library corresponding to 86% of the 19,000 predicted genes in *C. elegans *[5]. This library has already been screened for genes involved in a wide range of biological processes including development [6,7], ageing [8-10], cell biology [11,12] and neurobiology [13].

RNAi effects have now been detected in all eukaryote groups; phylogenetic analyses suggest that the main components of the RNAi machinery can be traced back to the last common ancestor of the eukaryotes [14]. Gene silencing by RNAi is the outcome of a complex co-ordinated process. Many of the essential proteins involved in this process have been identified. RNAi is initiated by the cleavage of exogenous dsRNA into short interfering RNAs (siRNAs) by a dsRNA-specific RNAse enzyme Dicer [15]. These double-stranded ~21 nucleotide siRNAs interact with, and activate, a multiprotein RNA-induced silencing complex (RISC) and the activated RISC then recognises and cleaves RNA molecules homologous to the interfering siRNA [16,17]. Gene silencing via RNAi can also be triggered by endogenous double stranded micro RNAs (miRNAs) which regulate gene expression at the post-transcriptional level in metazoans [18] and plants [19]. RNAi-related mechanisms are also involved in heterochromatin formation [20,21].

Although the core features of the RNAi response are conserved among eukaryotes, several taxon-specific variations occur. Taxa differ in their ability to take up dsRNA, to transmit siRNA systemically to other tissues and in the magnitude and duration of the RNAi response. While various cell types can efficiently take up dsRNA in *Drosophila melanogaster *[22], the response is cell autonomous and the flies are unable to produce a systemic response to the localized introduction of dsRNA [23]. Naturally occurring systemic transmission of siRNAs also appears to be absent from vertebrates. Other organisms including *C. elegans *[1], the insect parasitic nematode *Heterorhabditis bacteriophora *[24], the planarian worm *Schmidtea mediterranea *[25], the coelenterate *Hydra *[26], insect species including a grasshopper [27] and cockroach [28] and several plant species [29-32] have the ability to generate a systemic RNAi response. In *C. elegans *two genes *sid-1 *and *sid-2 *are required for the uptake of dsRNA from the intestine (sid = systemic RNAi-deficient) [33,34]. SID-1 is a multispan transmembrane protein which appears to function as a channel for the diffusion of dsRNA [33,35], and expression of nematode SID-1 in *Drosophila *cells which lack a *sid-1 *homolog resulted in the rapid import of dsRNA into the cells [35]. Although *sid-2 *mutants fail to show a systemic RNAi response to ingested dsDNA, the injection of dsRNA into the pseudocoel of these mutants generated a strong systemic RNAi response in the treated worms [34]. SID-2 encodes a single-pass transmembrane protein which is expressed in the membranes of the intestine lumen and although its mode of action has yet to be determined, SID-2 is an essential requirement for environmental RNAi in *C. elegans *[34].

Because of the complexity of the dsRNAi pathway, the loss of a specific RNAi component protein or the occurrence of mutations may lead to the lack of a systemic RNAi response. Exogenous dsRNAi function has been lost from specific lineages such as *Saccharomyces cerevisiae *[14] and *Trypanosoma cruzi *[36]. Lack of both a systemic and a cell-autonomous RNAi response to injected dsRNA was also observed in the free living nematodes *Oscheius tipulae *[37,38] and *Pristionchus pacificus *[39,40]. Testing of strains within the *Caenorhabditis *clade for susceptibility to RNAi by different delivery methods indicated that most strains are capable of a systemic RNAi response if dsRNA is delivered via microinjection, however six of the eight *Caenorhabditis *species tested, including *C. briggsae*, were resistant to dsRNAi via soaking or feeding [34,41]. When SID-2 from *C. elegans *was expressed in *C. briggsae *it conferred sensitivity to RNAi by soaking [34]. A naturally occurring strain of *C. elegans *from Hawaii was found to be resistant to RNAi by feeding or injection of germline-expressed genes and this defect was the result of mutations in the Argonaute gene *ppw-1 *[42]. The minimum defining feature of a RISC complex is that it contains one Argonaute family member and a small-RNA guide strand [43]. The Argonaute gene family contains 20 members in *C. elegans *[44], which suggests that several distinct RISC complexes may be formed in this organism. Resistance or partial resistance to RNAi has also been demonstrated in animal parasitic nematode species such as *Haemonchus contortus *[45] and *Ostertagia ostertagi *[46]. This resistance to RNAi may be due to difficulties in developing suitable *in vitro *culture conditions and delivery methods for these parasites, but the available data suggest that the RNAi pathway may not be fully functional in some animal parasitic nematodes [47]. By contrast, several plant parasitic nematode species are susceptible to RNAi, including the nematodes *Heterodera glycines *and *Globodera pallida *[48] and *Meloidogyne incognita *[49]. Viney and Thompson [50] propose that the limited success of RNAi in animal parasitic nematodes to date might be circumvented if, instead of the soaking protocols currently in use, the dsRNA were delivered to the worms' pseudocoel. Alternatively it may be necessary to genetically engineer animal parasitic nematodes to express the *sid-1 *and *sid-2 *genes from *C. elegans *[50].

Native *Drosophila *S2 cells lack a SID-1 homolog, however these cells become competent to take up dsRNA when exposed to brief serum starvation in the presence of dsRNA, followed by incubation in medium containing serum [22,51]. Systemic RNAi effects in *C. elegans *body wall muscles are also enhanced by starvation [33]. Observations in *C. elegans *and *Drosophila *suggest that environmental or physiological signals may be important in the uptake and spread of dsRNA. Using an RNAi approach Saleh *et al. *[52] have shown that dsRNA is taken up in serum starved *Drosophila *S2 cells by receptor mediated endocytosis and these authors demonstrated that a functional intracellular vesicle transport system is also required for systemic RNAi in *C. elegans*. It has been hypothesized that an RNAi response to exogenous dsRNA evolved as a defence against infectious transposons and viruses [53,54]. Many viruses encode viral suppressors of RNA silencing (VSRs) to counteract the hosts' defence against viral encoded dsRNAs [55]. VSRs target several of the key steps in the RNA silencing pathway and VSRs encoded by viruses of different families often share no homology at the primary amino acid sequence level [55]. Pharmacological evidence suggests that in serum starved *Drosophila *S2 cells dsRNA uptake is mediated by pattern recognition receptors of the scavenger-receptor family [52], a family of receptors with roles in the innate immune response [56]. The ability to mount a systemic RNAi response to ingested dsRNA is absent in many *Caenorhabditis *species and it is also poorly developed in animal parasitic nematodes, and both systemic and cell autonomous RNAi are absent in the nematodes *P. pacificus *and *O. tipulae*. In contrast *C. elegans, H. bacteriophora *and several species of plant parasitic nematodes are susceptible to RNAi from environmentally supplied dsRNA. This phenotypic variability among nematodes may result from differing niche-specific selective pressures affecting environmental RNAi and the innate immune response.

We have previously shown that the free living bacterial feeding nematode *Panagrolaimus superbus *is a suitable model for studies on the molecular basis of anhydrobiosis because of its remarkable ability to survive extreme desiccation [57]. As a bacterial feeder, *Panagrolaimus *can be readily cultured in the laboratory with *E. coli *as a food source, using methods developed for *C. elegans *[58]. We therefore investigated whether *Panagrolaimus *is susceptible to RNAi by feeding. Our results show that potent and specific RNAi effects can be achieved in *Panagrolaimus *by feeding it on *E. coli *expressing dsRNA for genes with essential conserved functions. This demonstration that *Panagrolaimus *is amenable to RNAi by feeding will allow the development of high throughput methods of RNAi screening for *P. superbus *and further enhance the utility of this nematode as a model system for the study of the molecular biology of anhydrobiosis, and as a possible satellite model nematode for comparative and functional genomics.

## Results and Discussion

### *Panagrolaimus *fed with *E. coli *expressing dsRNA for *C. elegans *embryonic lethal genes give rise to progeny with highly penetrant embryonic lethal phenotypes

**Figure 1:**
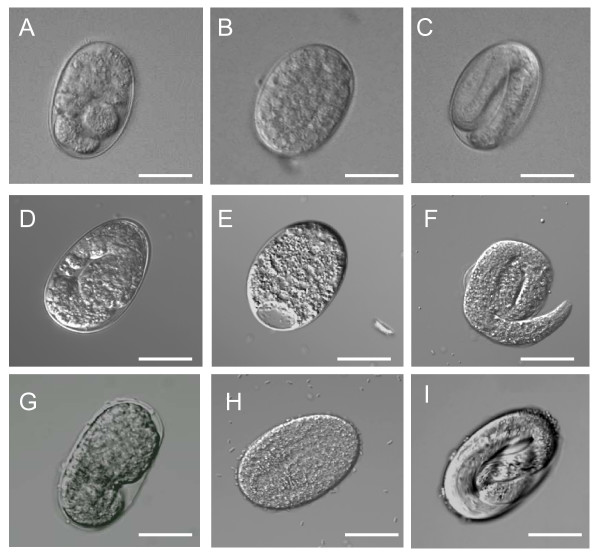
**The RNAi phenotypes obtained among the progeny of *Panagrolaimus *females fed on *E. coli *clones expressing dsRNA for the *Caenorhabaditis elegans *for the embryonic lethal genes *Ce-lmn-1 *and *Ce-ran-4***. (A) Embryo from a *P. superbus *female fed on *Ce-lmn-1*; (B) Embryo from a *P. superbus *female fed on *Ce-ran-4*; (C) Control embryo from *P. superbus *fed on *E. coli *containing the empty L4440 plasmid vector; (D) Embryo from a *Panagrolaimus *sp. PS1159 female fed on *Ce-lmn-1*; (E) Embryo from a *Panagrolaimus *sp. PS1159 female fed on *Ce-ran-4*; (F) Control embryo from *Panagrolaimus *sp. PS1159 fed on *E. coli *containing the empty L4440 plasmid vector; (G) Embryo from a *C. elegans *female fed on *Ce-lmn-1*; (H) Embryo from a * C. elegans *female fed on *Ce-ran-4*; (I) Control embryo from *C. elegans *fed on *E. coli *containing the empty L4440 plasmid vector. The scale bar represents 30 μM.

**Figure 2:**
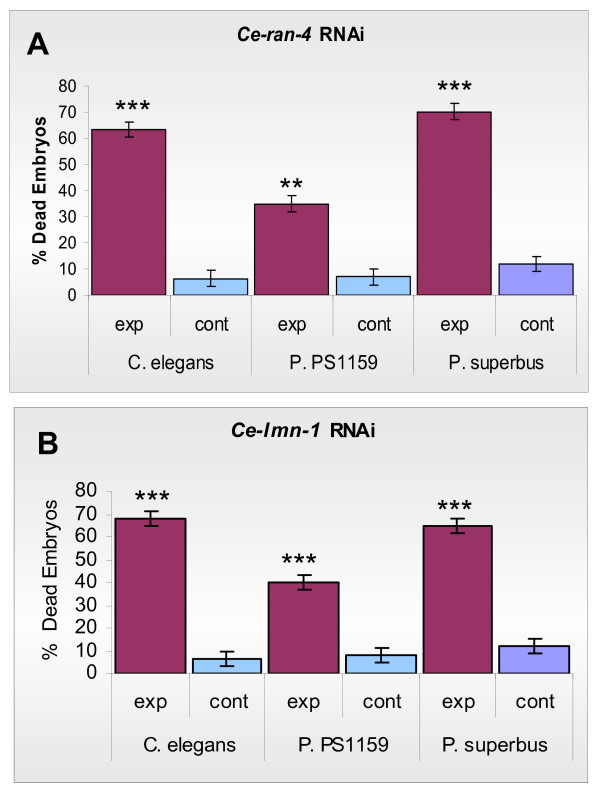
**The penetrance of the dsRNAi embryonic lethal phenotypes observed among the progeny of *C. elegans*, *P. superbus *and *Panagrolaimus *sp. PS1159 following ingestion of *E. coli *expressing dsRNA for the embryonic lethal genes *Ce-lmn-1 *and *Ce-ran-4***. (A) The percentage of embryonic lethal RNAi phenotypes observed in progeny of nematodes exposed to *Ce-ran-4*; (B) The percentage of embryonic lethal RNAi phenotypes observed in progeny of nematodes exposed to *Ce-lmn-1*. For both genes tested the control nematodes were fed on *E. coli *containing the empty L4440 plasmid vector. Data are presented as the mean and standard error of the mean of a minimum of 5 biological replicates, each replicate count containing a minimum of 50 embryos. ***P < 0.001; **P < 0.01 as compared to controls using a 2-tailed T test.

We tested two species of *Panagrolaimus *for RNAi effects using *E. coli *clones from a *C. elegans *feeding library [59]. The selected clones contained inserts from the genes *Ce-lmn-1 *(DY3.2) and *Ce-ran-4 *(R05D11.3). The functions of these genes are conserved among eukaryotes and they yield severe embryonic lethal RNAi phenotypes in *C. elegans *[59]. The *C. elegans *genome contains a single lamin gene, *lmn-1 *which encodes a nuclear lamin protein [60]. Previous RNAi studies have shown that *C. elegans lmn-1 (RNAi) *embryos have defects in chromatin organization, cell cycle progression and chromosome segregation [60] and that embryogenesis is halted before the embryo is enclosed by epidermal tissue [6]. The *Ce-lmn-1 (RNAi) Panagrolaimus *embryos show similar defects to those described for *C. elegans *and the terminal arrest phenotype is similar in both nematode genera (Figure 1A, 1D, 1G). *Ce-ran-4 *encodes an ortholog of nuclear transport factor 2 [61] which is responsible for importing the Ras-related nuclear protein RanGDP into the nucleus [62]. Ran is an essential component in the transport of many proteins and nucleic acids between the nucleus and the cytoplasm [63]. RNAi of *ran-4 *in *C. elegans *resulted in an embryonic lethal phenotype in which the embryos arrested as a multicellular mass (<200 cells) and the nuclear membranes were indistinct [6,61]. The terminal arrest phenotypes of *Ce-ran-4 (RNAi) *embryos in *Panagrolaimus *were also similar to those of *C. elegans *(Figure 1B, 1E, 1H). Although embryonic lethal RNAi phenotypes were observed for both species of *Panagrolaimus*, the RNAi effect showed greater penetrance in *P. superbus *than in *Panagrolaimus *sp. 1159 (Figure 2). From Figure 2 it can also be seen that the penetrance of the heterologous RNAi effect observed in *P. superbus *for both dsRNA constructs was comparable to penetrance of the RNAi phenotypes observed in *C. elegans *which had been treated with homologous dsRNA constructs. All three species tested (*C. elegans*, *Panagrolaimus *sp. PS1159 and *P. superbus*) showed significant differences in the percentage dead embryos between the experimental and control populations for both target genes tested (Two sample T-Test, P = 0.00).

### Embryonic lethal RNAi phenotypes obtained by feeding *Panagrolaimus *with *E. coli *expressing dsRNA for *Panagrolaimus *genes

**Figure 3:**
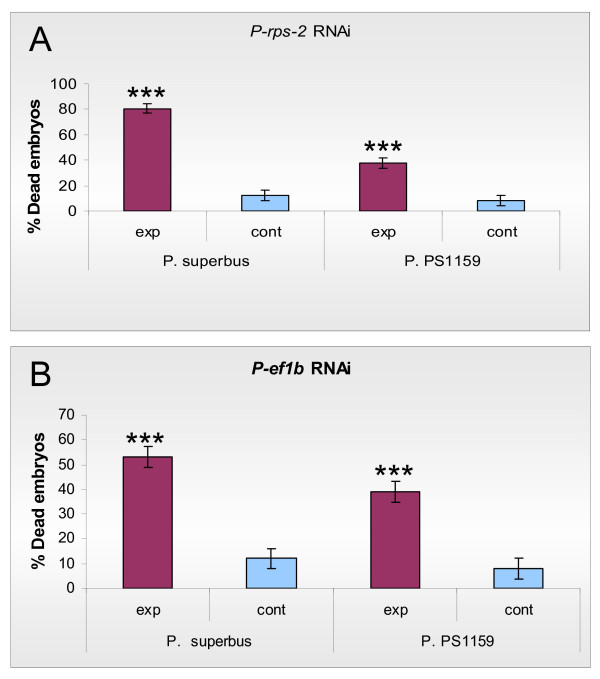
**The percentage of embryonic lethal RNAi phenotypes obtained by feeding *P. superbus *and *Panagrolaimus *sp. PS1159 on *E. coli *feeding clones expressing dsRNA for the *ef1b *and *rps-2 *genes from *Panagrolaimus *sp. PS1159**. (A) The percentage of embryonic lethal progeny observed among the progeny of female nematodes exposed to dsRNA for *P-rps-2*; (B) The percentage of embryonic lethal progeny observed in nematodes exposed to dsRNA for *P-ef1b*. For both genes tested the control nematodes were fed on *E. coli *containing the empty L4440 plasmid vector. Data are presented as the mean and standard error of the mean of a minimum of 5 biological replicates, each replicate containing a minimum of 20 embryos ***P < 0.001; **P < 0.01 as compared to controls using a 2-tailed T test.

*E. coli *feeding strains were constructed using sequences for two genes from *Panagrolaimus *sp. PS1159 whose orthologs have embryonic lethal RNAi phenotypes in *C. elegans*. These were the *elongation factor 1-beta (ef1b) *gene and the ribosomal protein small subunit S2 (*rps-2*) gene. The *Ce-rps-2 *gene demonstrated both a sterile maternal RNAi phenotype [64] and an embryonic lethal RNAi phenotype in *C. elegans *[65]. The *Panagrolaimus rps-2 *gene also yielded an embryonic lethal RNAi phenotype in both *Panagrolaimus *species tested (Figure 3A), with a significant difference in percentage dead embryos between the experimental and control populations in both cases (Two sample T-Test, P = 0.00). Here also the heterologous RNAi effect in *P. superbus *(80% embryonic lethality) was stronger than the homologous RNAi effect in *Panagrolaimus *sp. PS1159 (38% embryonic lethality). The average number of eggs laid per female nematode was compared for the control and RNAi treated *Panagrolaimus *populations and a Two sample T-Test (p = 0.62) did not reveal any significant difference in fecundity between the populations. The dsRNAi embryonic lethal phenotype for the *Panagrolaimus *sp. PS1159 *ef1b *gene also showed a stronger heterologous RNAi effect in *P. superbus *(53% embryonic lethality) than the homologous RNAi effect in *Panagrolaimus *sp. PS1159 (39% embryonic lethality), however there was a significant difference in the percentage of dead embryos between the experimental and control populations in both nematode species (Two sample T-Test, P = 0.00).

### Exposure of *Panagrolaimus *sp. PS1159 to homologous dsRNA by feeding results in a reduction of mRNA transcript levels for the target genes

**Figure 4:**
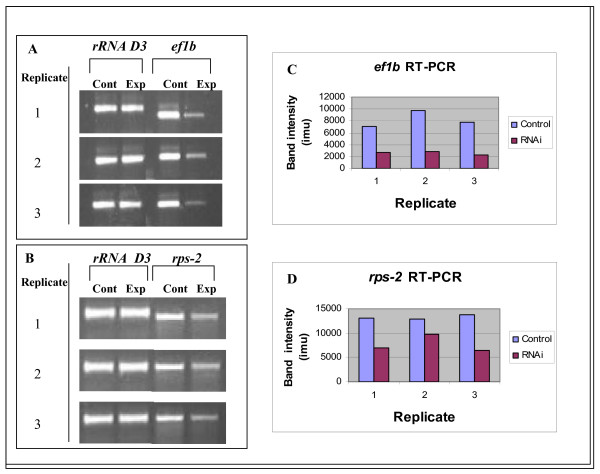
**Single nematode RT-PCR to assess transcript levels for the *P-ef1b *and *P-rps-2 *genes in control and RNAi treated *Panagrolaimus *sp. PS1159 nematodes.** RNA was isolated from individual nematodes whose progeny showed a positive RNAi phenotype and from individual control nematodes fed on *E. coli *containing the empty L4440 plasmid vector. Transcript levels were determined by RT-PCR for the target gene and for the *rRNA D3 *gene (which served as an internal control). (A) Transcript levels for the *P-ef1b *and *rRNA D3 *genes in individual *Panagrolaimus *sp. PS1159 adult females fed on *E. coli *expressing *P-ef1b *dsRNA (each of the three replicates corresponds to a separate nematode); (B) Transcript levels for the *P-rps-2 *and *rRNA D3 *genes in individual *Panagrolaimus *sp. PS1159 females fed on *E. coli *expressing *P-rps-2 *dsRNA (each of the three replicates corresponds to a separate nematode); (C, D) Quantification of the RT-PCR products for the *P-ef1b *and *P-rps-2 *genes in control and RNAi treated *Panagrolaimus *sp. PS1159 nematodes. The band intensities are given in ImageQuant™ machine units (imu).

Single nematode RT-PCR (Figure 4) showed that a reduction in mRNA transcript levels occurred for the target genes in the RNAi treated *Panagrolaimus *sp. PS1159 nematodes. For the *ef1b *gene the reduction in transcript level was 30% that of the controls while for the *rps-2 *gene the mRNA transcript levels were reduced to 55% of the controls (Figure 4C, D). There was no significant difference between the treated and control worms in the mRNA transcript levels for the D3 segment of the nuclear large subunit rRNA gene which was used as an internal control. This indicates that the dsRNAi effect in *Panagrolaimus *is specific for the targeted genes.

### Exposing *Panagrolaimus superbus *to homologous dsRNA for genes encoding contractile proteins results in potent and highly penetrant locomotor and growth defects

**Figure 5:**
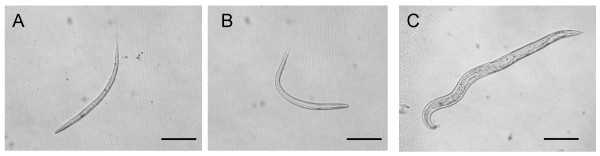
**Larval arrest phenotypes obtained by plating *P. superbus *eggs onto agar plates containing a lawn of *E. coli *expressing dsRNA for *P. superbus *genes encoding contractile proteins**. (A) dsRNAi phenotype for *Psu-ifb-1 *(cytoplasmic intermediate filament protein) and (B) dsRNAi phenotype for *Psu-act-2 *(actin-2). These images were taken 9 days post hatching. By this time the control embryos which had been plated onto agar plates containing a lawn of *E. coli *containing the empty L4440 plasmid vector had reached adulthood (C). The scale bar represents 100 μM.

*E. coli *feeding strains were constructed for two genes from *Panagrolaimus suprbus *whose *C. elegans *orthologs encode contractile proteins. These genes were *Psu-ifb-1 *and *Psu-act-2*. Each of these genes has multiple RNAi phenotypes in *C. elegans*. The *Ce-ifb-1 *gene encodes two isoforms of a cytoplasmic intermediate filament protein that are expressed in the hypodermal cells of *C. elegans*, where they form attachment structures which connect the muscles to the cuticle [66]. These attachment structures transmit the contractile forces generated by the body muscles across the hypodermis to the cuticle. In *C. elegans *both IFB-1 isoforms have roles in epidermal morphogenesis and muscle attachment [67]. *C. elegans *larvae exposed to *ifb-1 *RNAi by feeding displayed a paralysis phenotype in which the hypodermis becomes detached from the cuticle [68]. Exposure of *P. superbus *L1 larvae to *Psu-ifb-1 *dsRNA by feeding resulted in a larval arrest and paralysis phenotype (Figure 5A). The phenotype was highly penetrant and the treated larvae were severely paralysed. Microscopic observations revealed waves or ripples of muscle contraction moving from the anterior to the posterior of the immobile worms, indicating that the contractile forces generated by the muscles were not being transmitted to the cuticle.

The *C. elegans *genome encodes five highly conserved actin genes. ACT-1, -2, -3 and -4 all share 99% amino acid identity, while ACT-5, the most divergent, shares 93% identity with the other actin isoforms [69]. The *act-1, -2, -3 *and *-4 *genes function primarily in muscle and myofilament containing cells in *C. elegans *[69,70], while *act-5 *expression is restricted to cells which contain microvilli [71]. A variety of mutant and RNAi phenotypes have been associated with impared actin gene function in *C. elegans*, including defects in embryonic cell division [72], defects in muscle contractile function [73] including pharyngeal function [74], and growth defects [71]. *P. superbus *larvae grown on *E. coli *expressing *Psu-act-2 *dsRNA displayed severe larval lethal, larval arrest, and paralysed phenotypes (Figure 5B). The treated larvae were unable to grow beyond the first or second larval stages. The worms were immobile, although pharyngeal function appeared to be retained.

### *Panagrolaimus*: a potential satellite model nematode for functional genomics

Comparative studies show that extensive genetic diversity occurs within the Phylum Nematoda [75,76]. Consequently it is not always possible to extrapolate from the model nematode *C. elegans *to other members of the Phylum Nematoda; studies in additional representative nematode species are often necessary. Several additional species of plant and animal parasitic nematodes have been selected as model organisms. Comparative studies are also carried out on other species in the *Caenorhabditis *clade [76,77] and on the free-living bacterial feeding nematodes *Oeschius tipulae *[38,78] and *Pristionchus pacificus *[40,79]. Neither *O. tipulae *nor *P. pacificus *(both members of the suborder Rhabditina) display gene silencing in response to exogenous dsRNA. However injection of antisense morpholino DNA oligonucleotides into both of these satellite models results in a partial inactivation of the corresponding gene product [37] or in phenotypes with low penetrance [39]. Morpholino antisense oligonucleotides block mRNA translation when complementary to the 5' untranslated region of the target gene or to the first 25 bases downstream of the start codon, and they achieve their effect by preventing ribosomal binding [80]. The use of morpholinos presents difficulties for high-throughput gene knockout experiments because of the need for specific sequence information at the 5' end of the gene, the requirement for a means of delivery of the oligonucleotides to the target cells, and the lack of a systemic response.

**Figure 6:**
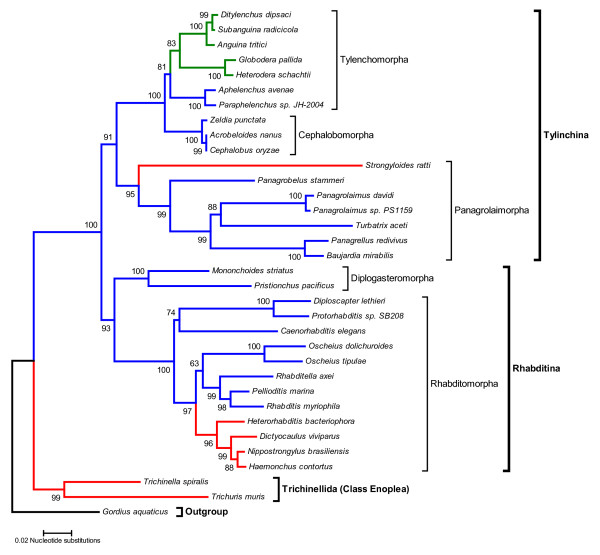
**The molecular phylogeny of selected members of the Order Rhabditida showing the phylogenetic relationships between *Caenorhabditis elegans *and other rhabditid and tylenchid nematodes**. Lineages in blue correspond to free-living nematodes, plant parasite lineages are green and animal parasite lineages are red. This Minimum Evolution tree [92] was constructed using 18S small subunit rRNA gene sequences. Bootstrap values (10,000 replicates) are placed next to the nodes.

We have previously shown that *Panagrolaimus *is a suitable model for studies on the molecular basis of anhydrobiosis [57]. Panagrolaimid nematodes occupy a diversity of niches ranging from polar, temperate and semi-arid soils to terrestrial mosses. Thus many *Panagrolaimus *species are adapted to tolerate freezing and desiccation [81-83]. Our current data show that when fed on *E. coli *expressing dsRNA targeting *Panagrolaimus *genes the nematode mounts a potent and specific systemic RNAi response. This makes *Panagrolaimus *an ideal system with which to carry out high-throughput RNAi screens for studies on the molecular basis of anhydrobiosis and cryobiosis. *Panagrolaimus *is a member of the suborder Tylenchina of the Order Rhabditida and thus it is more distantly related to *C. elegans *than *Oeschius *or *Pristionchus *(Figure 6). The Suborder Tylenchina contains three major lineages [84]: the Panagrolaimorpha which includes the free living bacterial feeding panagrolaimids as well as the zooparasitic strongyloids and steinernematids; the Cephalobomorpha, bacterial feeding nematodes that, like the panagrolaimaids, have a widespread distribution and are capable of surviving in extremely dry and cold environments and the Tylenchomorpha, a group of plant parasites and fungal feeders which frequently has anhydrobiotic encysted infective stages (e.g. *Globodera*, *Meliodogyne*), as well as species which can enter anhydrobiosis throughout most or all of their life cycle (e.g. *Aphelencus avenae*). Within the genus *Panagrolaimus *several changes in reproductive mode have occurred. Examples of gonochoristic (male female), hermaphroditic (self-fertilization), and parthenogenetic (development of unfertilized eggs) reproduction have been reported [85]. Thus *Panagrolaimus *is an excellent model system to study the evolutionary origins and developmental consequences of parthenogenetic reproduction. Being closely related to the zooparasitic strongyloids and steinernematids and the plant parasitic tylenchid nematodes, *Panagrolaimus *could also be used in comparative genomic approaches to study the evolution of parasitic lifestyles.

## Conclusion

Our results show that potent and specific RNAi effects can be achieved in two species of *Panagrolaimus *by feeding them on *E. coli *expressing dsRNA for genes with essential conserved functions. RNAi was first described in the nematode *C. elegans*, however some nematode species are refractory to RNAi, including *O. tipulae *[37,38], *P. pacificus *[39], several species of *Caenorhabdtis *[34,41] and some animal parasitic nematodes such as *H. contortus *[45] and *O. ostertagi *[46]. In contrast many species of plant parasitic nematodes have potent RNAi responses to exogenous RNAi [48,49]. Here we show that two species of *Panagrolaimus *are amenable to RNAi by feeding. *Panagrolaimus *is a soil dwelling bacterial feeding nematode, as are *C. elegans*, *P. pacificus *and *O. tipulae*, but as a member of the suborder Tylenchina *Pangarolaimus *is more distantly related to *C. elegans *than *Oeschius *or *Pristionchus*. Thus our data identify another nematode infraorder which is amenable to RNAi and provide additional information on the diversity of RNAi phenotypes in nematodes.

Panagrolaimaid nematodes occupy a diversity of niches ranging from polar, temperate and semi-arid soils to terrestrial mosses. Thus many *Panagrolaimus *species are adapted to tolerate freezing and desiccation [57,81-83]. Within the genus *Panagrolaimus *several changes in reproductive mode have occurred. Thus *Panagrolaimus *is an excellent model system to study the evolutionary origins and developmental consequences of parthenogenetic reproduction. Our demonstration that *Panagrolaimus *is amenable to RNAi by feeding will allow the development of high throughput methods of RNAi screening for *P. superbus*. This greatly enhances the utility of this nematode as a model system for the study of the molecular biology of anhydrobiosis and cryobiosis and as a possible satellite model nematode for comparative and functional genomics.

## Methods

### Source and culturing of *Panagrolaimus sp*. and *Caenorhabditis elegans*

The nematode strains *C. elegans *N2, and *Panagrolaimus *sp. PS1159 were obtained from the *Caenorhabditis *Genetics Centre (CGC). *P. superbus *(strain DF5050) was obtained from Prof. Bjorn Sohlenius, Swedish Museum of Natural History, Stockholm. The nematodes were cultured on NGM agar plates [57].

### *E. coli *feeding clones

Two *E. coli *RNAi feeding clones from the *C. elegans *chromosome I feeding library [59] were kindly provided by Dr. Julie Ahringer, Department of Genetics, University of Cambridge. These clones contained inserts from the embryonic lethal genes *Ce-ran-4 *(R05D11.3) and *Ce-lmn-1 *(DY3.2). Bacterial feeding strains were constructed for the *Panagrolaimus *genes using the L4440 plasmid vector [4] and *Escherichia coli *HT115(DE3) cells as described by Kamath and Ahringer [5]. The *Panagrolaimus *genes were selected from a panel of expressed sequence tags (ESTs) which we have generated for *P. superbus *and *Panagrolaimus *sp. PS1159 (Tyson *et al. *unpublished). The target genes were selected based on their gene identity as determined by BLAST analysis [86] and the RNAi phenotypes of their orthologs in *C. elegans*, as listed in WormBase [87]. The accession numbers of the *Panagrolaimus *ESTs and their cDNA fragment sizes are as follows: *ef1b*, EU368942 (625 bp); *rps-2*, EU368941 (649 bp); *ifb-1*, EU368943 (360 bp) and *act-2*, EU368944 (391 bp). Gene fragments of the *ef1b *and *rps-2 *genes for insertion into the L4440 plasmid were prepared by PCR using the gene specific primer pairs EF1 Forward/EF1 Reverse and RPS Forward/RPS Reverse (The sequences of these PCR primers are listed in Additional File 1). Gene fragments corresponding to the entire EST sequences of the *ifb-1 *and *act-2 *genes were prepared from the plasmid name using M13 F and R primers and cloned into the L4440 vector. A separate control experiment (data not presented) showed that an RNAi feeding clone containing dsRNA for the multiple cloning site of the pDNR-LIB plasmid had no observable phenotype when fed to *P. superbus *or *Panagrolaimus *sp. PS1159.

### RNAi feeding protocol

NGM agar plates (9 cm) containing 1 mM iso-propyl-β-D-thiogalactopyranoside (IPTG), 50 ug/ml ampicillin (Amp) and 12.5 ug/ml tetracycline (Tet) were prepared. The plates were allowed to dry inverted for ~4 days at room temperature. They were then were spread with cells from an overnight *E. coli *culture (strain HTT1(DE3)) containing the empty L4440 plasmid (control) or with *E. coli *HTT1(DE3) transformed with an L4440 plasmid containing the target gene fragment inserted into the multiple cloning site, and flanked on both sides by an IPTG-inducible T7 RNA polymerase promoter [4]. The plates were grown at 20°C overnight to allow a lawn of *E. coli *to form and to begin induction and expression of dsRNA. Axenised eggs were prepared from gravid female nematodes by bleaching them with hypochlorite [88]. The axenised eggs were rinsed three times in S Basal buffer [58], then aliquotted onto NGM agar feeding plates. The plates were incubated at 20°C and monitored daily for the presence of visible RNAi phenotypes in the treated nematodes. To analyse embryonic lethal RNAi phenotypes young L4 stage worms were rinsed from the treatment and control plates using sterile S Basal buffer and approximately 5 worms were transferred to each of 5–8 replicate 3 cm NGM/IPTG/Amp/Tet agar plates containing a lawn of either control *E. coli *or *E. coli *expressing dsRNA for the target gene. These worms were allowed to grow to adulthood and lay eggs for 24 hours and they were then transferred to a second set of replicate plates. The worms were again allowed to lay eggs for 24 hours before being transferred to a third set of plates. The worms were allowed to lay eggs on the third set of plates until laying ceased and the adult worms were then removed from the plates. The number of dead embryos on each of the three sets of plates was determined by microscopic observation over a number of days for both the control and experimental treatments. Standard deviation and standard errors were calculated using the individual counts of surviving nematodes and not on proportional data, but in the histograms survival data are presented as percentage survival.

To observe possible embryonic lethal RNAi phenotypes synchronous eggs from control and RNAi treated cultures were rinsed from the agar surface using S Basal buffer and centrifuged at 3,000 g for five minutes. The concentrated eggs were transferred to a glass slide and gently covered with a glass cover slip. Embryos were observed using an Olympus EX51 microscope fitted with a U-CMAD3 imaging system and an Olympus U-RFL-T camera.

### RT-PCR analysis of gene expression levels

Individual adult female nematodes whose progeny displayed an embryonic lethal RNAi phenotype were used for RT-PCR. RNA was isolated from single nematodes using the Absolutely RNA Microprep Kit (Stratagene), which also included a DNAse treatment prior to the reverse transcription step to remove any genomic DNA (gDNA) contamination. As a control, PCR was attempted on each sample after DNAse treatment and prior to RT-PCR to ensure all gDNA had been removed. PCR samples were then analysed by spectrophotometry and agarose gel electrophoresis and samples showing no gDNA amplification were chosen for RT-PCR experiments. Each worm RNA preparation yielded 30 μl of extracted RNA. Six μl of the extracted RNA solution was used for each RT-PCR reaction. RT-PCR was carried out using the QuanTitect SYBR Green RT-PCR kit (Qiagen). The upstream test forward primers used for RT-PCR analysis targeted a region not included in the dsRNA feeding construct. The primers used were the EF1-F-TEST and the EF1 reverse primers for the *ef1b *gene and the RPS-F-TEST and the RPS reverse primers for the *rps*-2 gene. The sequences of all PCR primers are listed in Additional File 1. RT-PCR was also carried using the D3 expansion region of the ribosomal RNA gene as an internal control gene for each single worm RNA preparation. Amplification of the rRNA D3 domain was carried out as described previously [57]. The RT-PCR conditions were as follows: 94°C for 5 min at the start of the reaction was followed by 30 minutes at 50°C to allow reverse transcription of the RNA. The PCR cycling conditions were as follows: 94°C, 1 min; 55°C, 1.5 min; 72°C, 2 min. To obtain PCR product during the linear phase of the PCR reaction a 10 μl aliquot from the PCR reaction mixture was sampled at 20, 25, 30 and 35 cycles for each gene used. The products of the RT-PCR reactions were visualised on 1% agarose gels with 10 μl of each RT-PCR product loaded per gel. Image analysis of the RT-PCR gels was carried out and band intensities were measured in machine units using the ImageQuant™ TL software package from GE Healthcare.

### Phylogenetic analyses

Nucleotide sequences for the 18S small subunit rRNA nuclear gene of 34 taxa were downloaded from the NCBI database [89]. The accession numbers for these sequences are given in Additional File 2. The sequences were aligned using the ClustalW algorithm as implemented in the MEGA software package (version 3.1) [90]. The alignment was manually edited by removing poorly aligned blocks at the 5' and 3' ends and ambiguously aligned internal segments. The final alignment contained 1,275 aligned characters. Phylogenetic trees were constructed using the Neighbor-Joining method [91] and Minimum Evolution method [92] as implemented by MEGA version 3.1. The tree topologies recovered trees by both methods were very similar, differing only in the placement of the *Oscheius *clade (see Figure 6 and Additional File 3).

### Statistical analyses

All statistical analyses were carried out using Minitab™ version 14.1 (Minitab Inc., State College, Pennsylvania, USA). The 2-tailed T-Test was used to test for difference between populations. The null hypothesis that population means are all equal was tested at the *P *= 0.05 level of significance. T-Tests were carried out using the individual counts of surviving nematodes and not on proportional data.

## Authors' contributions

AJS and TT did the RNAi experiments. ID selected and tested the *C. elegans *feeding constructs. ID and AJS carried out the microscopy. AJS did the RT-PCR experiments. JB selected and prepared the *Panagrolaimus *sp. PS1159 cDNA sequences. AMB did the phylogenetic reconstruction. AMB, AJS, TT and ID designed the study and AMB coordinated the study. AJS and AMB drafted the manuscript. All authors read and approved the final manuscript.

## Supplementary Material

Additional file 1 **Table 1**. Sequences of the PCR primers used for single nematode RT-PCR. Click here for file

Additional file 2 **Table 2**. Accession Numbers of the small subunit ribosomal RNA sequences used to construct a molecular phylogeny of selected members of the Order Rhabditida. Click here for file

Additional file 3 **Additional figure**. The molecular phylogeny of selected members of the Order Rhabditida showing the phylogenetic relationships between Caenorhabditis elegans and other rhabditid and tylenchid nematodes.  Lineages in blue correspond to free-living nematodes, plant parasite lineages are green and animal parasite lineages are red.  This Neighbor-Joining tree [91] was constructed using 18S small subunit rRNA gene sequences.  Bootstrap values (10,000 replicates) are placed next to the nodes. Click here for file
