# Supplementary material for: Systemic RNAi mediated gene silencing in the anhydrobiotic nematode Panagrolaimus superbus
Source: BMC Mol Biol. 2008 Jun 19;9:58. doi: 10.1186/1471-2199-9-58 (PMC2453295; doi:10.1186/1471-2199-9-58)
Supplement: Additional file 1 — Table 1. Sequences of the PCR primers used for single nematode RT-PCR. [file 1471-2199-9-58-S1.doc]

**Table 1**.

Sequences of the PCR primers used for single nematode RT-PCR

| **Primer** | **Sequence** |
| --- | --- |
| EF1 Forward | 5` TACAATCAATGCTGGCATCC 3` |
| EF1 Reverse | 5` CAGTTCCCCAGGTTAATCCA 3` |
| EF1 Test Forward | 5` TGCCTTTGAAGATCTTGTTC 3` |
| RPS Forward | 5` TGGTCATGTTGGTCTTGGTG 3` |
| RPS Reverse | 5` GACGAACAGGTGCTGTTGAA 3` |
| RPS Test Forward | 5` CGATTTCCTTTCAACTTCAAC 3` |
| rDNA D3A (Nunn *et al.,* 1996) | 5` GACCCGTCTTGAAACACGGA 3` |
| rDNA D3B (Nunn *et al.,* 1996) | 5` TCGGAAGGAACCAGCTACTA 3` |

**Reference**

Nunn, GB; Theisen, BF; Christensen, B; Arctander, P. **Simplicity-correlated size growth of the nuclear 28S ribosomal RNA D3 expansion segment in the crustacean order Isopoda.** *J Mol Evol* 1996, **42**: 211-223.
