# Supplementary material for: Systemic RNAi mediated gene silencing in the anhydrobiotic nematode Panagrolaimus superbus
Source: BMC Mol Biol. 2008 Jun 19;9:58. doi: 10.1186/1471-2199-9-58 (PMC2453295; doi:10.1186/1471-2199-9-58)
Supplement: Additional file 2 — Table 2. Accession Numbers of the small subunit ribosomal RNA sequences used to construct a molecular phylogeny of selected members of the Order Rhabditida. [file 1471-2199-9-58-S2.doc]

Table 2. Accession Numbers of the small subunit ribosomal RNA sequences used to construct a molecular phylogeny of selected members of the Order Rhabditida**.**

| **Nematode species** | **Accession number** |
| --- | --- |
| *Acrobeloides nanus* | DQ102707 |
| *Anguina tritici* | AY593913.1 |
| *Aphelenchus avenae* | AF036586 |
| *Baujardia mirabilis* | AF547385.1 |
| *Caenorhabditis elegans*, N2 | X03680 |
| *Cephalobus oryzae*, strain PS1165 | AF034390 |
| *Dictyocaulus viviparus* | AJ920361.1 |
| *Diploscapter coronatus* strain DiScCor | AY593921 |
| *Ditylenchus dipsaci* | AY593911.1 |
| *Globodera pallida* | AF036592 |
| *Gordius aquaticus* (outgroup) | X87985 |
| *Haemonchus contortus* | L04153 |
| *Heterodera schachtii* | AY284617.1 |
| *Heterorhabditis bacteriophora* | AF036593 |
| *Mononchoides striatus* | AY593924.1 |
| *Nippostrongylus brasiliensis* | AF036597 |
| *Oscheius dolichuroides* | AF082998 |
| *Oscheius tripulae*, strain CEW1 | AF036591 |
| *Panagrellus redivivus*, strain PS1163 | AF083007 |
| *Panagrobelus stammeri* | AF202153 |
| *Panagrolaimus davidi* | AJ567385.1 |
| *Panagrolaimus sp*., strain PS1159 | U81579 |
| *Paraphelenchus sp.* JH-2004 | AY284642.1 |
| *Pellioditis marina,* strain SB178 | AF083021 |
| *Pristionchus pacificus*, strain PS312 | AF083010 |
| *Protorhabditis sp.* SB208 | AF083024 |
| *Rhabditella axei*, strain DF5006 | U13934 |
| *Rhabditis myriophila,* strain DF5020 | U81588 |
| *Strongyloides ratti* | U81581 |
| *Subanguina radicicola* | AF202164 |
| *Trichinella spiralis* | U60231 |
| *Trichuris muris* | AF036637 |
| *Turbatrix aceti* | AF202165 |
| *Zeldia punctata* | U61760 |
