# Supplementary material for: Systemic RNAi mediated gene silencing in the anhydrobiotic nematode Panagrolaimus superbus
Source: BMC Mol Biol. 2008 Jun 19;9:58. doi: 10.1186/1471-2199-9-58 (PMC2453295; doi:10.1186/1471-2199-9-58)
Supplement: Additional file 3 — Additional figure. The molecular phylogeny of selected members of the Order Rhabditida showing the phylogenetic relationships between Caenorhabditis elegans and other rhabditid and tylenchid nematodes. Lineages in blue correspond to free-living nematodes, plant parasite lineages are green and animal parasite lineages are red. This Neighbor-Joining tree [91] was constructed using 18S small subunit rRNA gene sequences. Bootstrap values (10,000 replicates) are placed next to the nodes. [file 1471-2199-9-58-S3.doc]

**Additional Figure. The molecular phylogeny of selected members of the Order Rhabditida showing the phylogenetic relationships between *Caenorhabditis elegans* and other rhabditid and tylenchid nematodes.**  Lineages in blue correspond to free-living nematodes, plant parasite lineages are green and animal parasite lineages are red. This Neighbor-Joining tree [91] was constructed using 18S small subunit rRNA gene sequences. Bootstrap values (10,000 replicates) are placed next to the nodes.
